# Descending Inputs to Spinal Circuits Facilitating and Inhibiting Human Wrist Flexors

**DOI:** 10.3389/fnhum.2018.00147

**Published:** 2018-04-17

**Authors:** Stefane A. Aguiar, Stuart N. Baker

**Affiliations:** Institute of Neuroscience, Newcastle University Newcastle upon Tyne, United Kingdom

**Keywords:** corticospinal tract, reticulospinal tract, H-reflex, Ib afferents, transcranial magnetic stimulation, click sound stimulation

## Abstract

Recently we reported in humans that electrical stimulation of the wrist extensor muscle extensor carpi radialis (ECR) could facilitate or suppress the H reflex elicited in flexor carpi radialis (FCR), for inter-stimulus intervals (ISIs) of 30 ms or 70 ms, respectively. The facilitation at 30 ms may be produced by both flexor afferents and extensor Ib afferents acting on a spinal circuit; the origin of the suppression at 70 ms is less certain. In this study, we investigated possible descending inputs to these systems. We used magnetic stimulation of the contralateral primary motor cortex, and click sound stimulation, to activate the corticospinal and the reticulospinal tracts respectively, and measured the effects on the H reflex conditioned by ECR stimulation. Corticospinal inputs reduced both the 30 ms facilitation and 70 ms suppression, indicating corticospinal inhibition of both circuits. By contrast, we failed to show any effect of clicks, either on the H reflex or on its modulation by ECR stimulation. This suggests that click-activated reticulospinal inputs to these circuits may be weak or absent.

## Introduction

Although assessing the function of spinal circuits non-invasively in humans can be a challenging task, several spinal circuits as well as ways of assessing them have been described in the literature—e.g., reciprocal inhibition (Day et al., 1984), recurrent inhibition (Bussel and Pierrot-Deseilligny, 1977), cutaneomuscular reflexes (Jenner and Stephens, 1982), amongst others. The investigation of spinal circuitry is crucial not only to understand function but to explore and possibly treat pathology. Despite the great progress achieved, one important gap was assessment of Ib pathways in human upper limb. A previously-proposed and promising method suggested that Ib circuits could be assessed by recording responses to electrical stimulation over the finger extensor tendons (Burne and Lippold, 1996); however, subsequent work appeared to indicate that these responses were instead generated by group III afferents (Priori et al., 1998). Cavallari et al. (1985) proposed that the action of Ib fibers from wrist extensors could be detected on the wrist flexor H reflex following radial nerve stimulation. However, this effect was not consistent between subjects, and was superimposed on the more pronounced suppression produced by Ia afferents.

Recently, we described an approach which allows a straightforward and easy assessment of Ib function in humans (Aguiar and Baker, 2018). The assessment consists of the measurement of the flexor carpi radialis (FCR) H-reflex conditioned by electrical stimulation of the extensor carpi radialis (ECR) muscle. At a 30 ms interval (ECR stimulation preceding median nerve), the FCR H-reflex is facilitated, with contributions from both wrist flexor and extensor Ib afferents via a putative spinal circuit. With a 70 ms interval, the FCR H-reflex is inhibited, but the specific components of the circuit generating this inhibition are still to be uncovered (Aguiar and Baker, 2018).

The primate hand is under sophisticated descending control, dominated by the corticospinal tract (CST), which is responsible for the production of fine fractionated digit movements (Lawrence and Kuypers, 1968; Porter and Lemon, 1993). Other descending pathways are also involved in hand function; recent work has demonstrated a role for the reticulospinal tract (Davidson and Buford, 2006; Baker, 2011). Neurons in the reticular formation modulate their discharge during fine finger movements at least as much as those in the motor cortex (Soteropoulos et al., 2012). Although both corticospinal and reticulospinal tracts make monosynaptic connections to motoneurons in primates (Phillips and Porter, 1977; Riddle et al., 2009), they also provide many inputs to spinal interneurons in the intermediate zone (Kuypers, 1981). Many spinal cord interneurons receive convergent inputs from the corticospinal and reticulospinal tracts, although selective input from only one descending tracts is also possible (Riddle and Baker, 2010). In humans, some of the best characterized spinal circuits are those responsible for reciprocal inhibition. It is possible to demonstrate descending inputs to these circuits using non-invasive cortical stimulation. Corticospinal inputs activated by transcranial magnetic stimulation (TMS) can enhance pre-synaptic inhibition of wrist flexors, although in the lower limb the opposite effect is seen, with corticospinal activation *reducing* pre-synaptic inhibition (Meunier and Pierrot-Deseilligny, 1998; Meunier, 1999). Corticospinal inputs can also reduce di-synaptic inhibition in wrist flexors (Mercuri et al., 1997).

In this study, we investigated whether the circuits producing facilitation and suppression of the FCR H-reflex following stimulation of the ECR muscle receive descending input. We used loud click sounds to activate the reticular formation (Fisher et al., 2012), and found no effect on either the circuit responsible for FCR facilitation or suppression. By contrast, TMS to primary motor cortex appeared to reduce both the facilitation and suppression of FCR generated by ECR stimulation, suggesting that both of these circuits are inhibited by corticospinal input.

## Materials and Methods

Fifteen healthy adults, 18–56 years of age, participated in this study (12 females, 3 males). This study was carried out in accordance with the recommendations of the ethics guidelines, Ethical Committee of the Medical Faculty, Newcastle University. The protocol was approved by the Ethical Committee of the Medical Faculty, Newcastle University. All subjects gave written informed consent in accordance with the Declaration of Helsinki.

The FCR H-reflex in the right arm was measured, evoked by stimulation of the median nerve at the cubital fossa (monophasic pulse, intensities up to 9.5 mA, 500 μs pulse width), and conditioned by electrical stimulation of the ECR muscle at 3× motor threshold (MT; monophasic pulse, intensities up to 24 mA, 1 ms pulse width). All procedures were as described previously, including electrode placement, and equipment for EMG recording and muscle and nerve electrical stimulation (Aguiar and Baker, 2018). The ECR intensity of 3× MT was chosen as it yields a robust effect on the H reflex, but this is not saturated—in our previous work, further facilitation could be produced by combining the ECR conditioning with a cutaneous stimulus. Two intervals between ECR conditioning stimulation and median nerve shock (ECR-Median nerve interval) were used in the study, 30 and 70 ms (ECR preceding median nerve). The FCR H-reflex conditioned by ECR stimulation was further conditioned by either TMS or click sounds, in two separate set of experiments. Some subjects participated in multiple protocols. All results obtained from a given protocol were included in the averaged results presented here.

### TMS Experiments

For TMS experiments, we used a Magstim 2002 stimulator with figure of eight coil (7 cm outer winding diameter; The Magstim Company Ltd, Whitland, UK), and first located the optimal site over left primary motor cortex to elicit a motor evoked potential (MEP) in the FCR muscle. Coil orientation was at a 45° angle to the midline, with the handle directed posteriorly; this produces current in the brain in a posterior-anterior direction. We then measured the passive threshold, defined as the minimal TMS intensity capable of producing a MEP in the FCR muscle with peak-peak amplitude >50 μV in 5 out of 10 measurements with the muscle at rest (4 s inter-stimulus interval, ISI). TMS intensity was set as 90% of this passive threshold. Different ISIs between TMS and median nerve stimulation were tested. Ten repetitions of each ISI were recorded with and without ECR conditioning stimulation. Twenty repetitions were recorded of the control H-reflex, with no conditioning stimulation, and 20 repetitions were also recorded of the H-reflex conditioned by ECR stimulation alone (with no TMS). H-reflex amplitudes were expressed as percentages of control H-reflex (with no conditioning stimulation). Intervals of 4 s were used in between H-reflex measurements to avoid homosynaptic depression. This entire procedure was repeated for each ECR-Median nerve interval tested (30 and 70 m) in random order. The ISIs tested with 30 ms ECR-Median nerve interval were −4, −3, −2, −1, 0, 1, 2, 3, 4 and 5 ms, and for the 70 ms ECR-Median nerve interval were −3, −2, −1, 0, 1, 5, 10, 15, 20, 25, 30, 35, 40 and 45 ms (negative intervals correspond to median nerve preceding TMS). For the 30 ms ECR-Median nerve interval, which causes facilitation of the FCR H-reflex, we decreased the intensity of median nerve stimulation so that the amplitude of the H-reflex + ECR matched the amplitude of the control H-reflex, with no conditioning stimulation. For the 70 ms interval, which causes FCR H-reflex inhibition, we increased the intensity of the median nerve shock so that the amplitude of the H-reflex + ECR matched the amplitude of the control H-reflex, with no conditioning stimulation. This size matching was confirmed through *t*-tests comparing the 20 repetitions of the control H-reflex, with no conditioning stimulation, and the 20 repetitions of the H-reflex conditioned by ECR stimulation alone (with no TMS). Only data sets in which no significant difference was detected in the *t*-tests were considered for analysis.

The peak-to-peak size of the H reflex was measured in each condition. Measurements of H reflex conditioned by TMS were expressed as a percentage of the unconditioned H reflex amplitude. Measurements of the H reflex conditioned by ECR stimulation and TMS were expressed as a percentage of the amplitude of the H reflex conditioned by ECR stimulation alone.

Nine subjects in total participated in the 30 ms ECR-Median nerve interval protocol. Given the fine-grain resolution of ISI for this protocol, we expressed the ISIs relative to the first ISI which showed a significant effect of TMS on the H-reflex. The interval of this first effect was described as 0 ms for all subjects and previous and subsequent intervals were adjusted accordingly and named early facilitation delays (EFDs). This meant that a different number of subjects contributed to each one of the intervals (EFDs). In the figures, we display data with EFDs −5, −4, −3, −2, −1, 0, 1, 2, 3, 4, 5, 6, 7, 8 and 9 ms. After this synchronization, statistical analysis was conducted across subjects. The first analysis had the purpose of investigating the effects of TMS alone on H-reflex measurements. This analysis therefore used only sweeps with no conditioning ECR stimulation. We first performed a two-way analyses of variance (ANOVA) with factors subjects and ISIs to investigate if TMS at different ISIs had any effect on H-reflex measurements. If the ANOVA showed a significant effect of ISI we then computed *t*-tests with the reference value of 100% (control H-reflex) to show which ISIs were affected by TMS. The second analysis aimed to compare results with TMS alone and with TMS + ECR conditioning stimulation. We first performed a three-way ANOVA with factors subjects, ISIs and with/without ECR conditioning stimulation to investigate if both the ISIs and the ECR conditioning stimulation had any effect on the H-reflex measurements. If the ANOVA showed significant effect of ECR conditioning stimulation and ISIs, we then used *t*-tests comparing the results from all subjects on each ISI with and without ECR stimulation. For ANOVA and *t*-tests the significance limit was set at *P* < 0.05.

For the protocol with ECR-Median nerve interval of 70 ms, we averaged results from nine subjects in each ISI. No synchronization was applied for this protocol given the longer ISIs tested, which dwarfed the small variations in EFD across subjects. Statistical analysis was conducted as described for the 30 ms ECR-Median nerve interval, to analyze both the effect of TMS alone on the H-reflex and the effect on ECR conditioning stimulation.

Findings from a single subject are illustrated in “Results” section. Statistical analysis of single subject data used measurements of H-reflex amplitude taken from single sweeps (rather than averages), and applied the same statistical tests as described above, except that ANOVA without the factor “subjects” was used. This was carried out only for the purposes of providing a visual indication on the graphs of whether points differed significantly from the unconditioned responses.

### Click Experiments

Click sounds were generated by delivering a 0.1-ms-wide, square excitation pulse into headphones, with a Z-weighted intensity A_Z_ of 125 dB SPL (amplifier Topaz SR20, Cambridge Audio, UK, driven with 5V input pulses with volume turned to maximum). Click sound stimulation was given to the left ear while responses were recorded from the FCR muscle in the right forearm. Similar to TMS experiments we also tested the same two ECR-Median nerve intervals of 30 and 70 ms and different ISIs between click sound and median nerve stimulation. The ISIs tested with 30 ms ECR-median nerve interval were −1, 0, 1, 2, 3, 4, 5, 6, 7, 8, 9, 10 and 11 ms, and for the 70 ms ECR-median nerve interval were −1, 0, 1, 2, 5, 10, 15, 20, 25, 30, 35, 40 and 45 (negative intervals correspond to median nerve preceding click). The number of repetitions in each ISI, control H-reflex and H-reflex conditioned by ECR stimulation alone was also the same used for TMS experiments. The matching of the sizes of H-reflex control and H-reflex conditioned by ECR alone (at both 30 and 70 ms ECR-Median nerve intervals) was conducted exactly as for TMS experiments. Statistical analyses were also conducted in the same way as for TMS experiments, except that no synchronization of ISIs relative to EFD was applied. Six and eight subjects participated in the 30 and 70 ms ECR-Median nerve interval protocols, respectively.

## Results

### TMS Experiments

**Figure 1:**
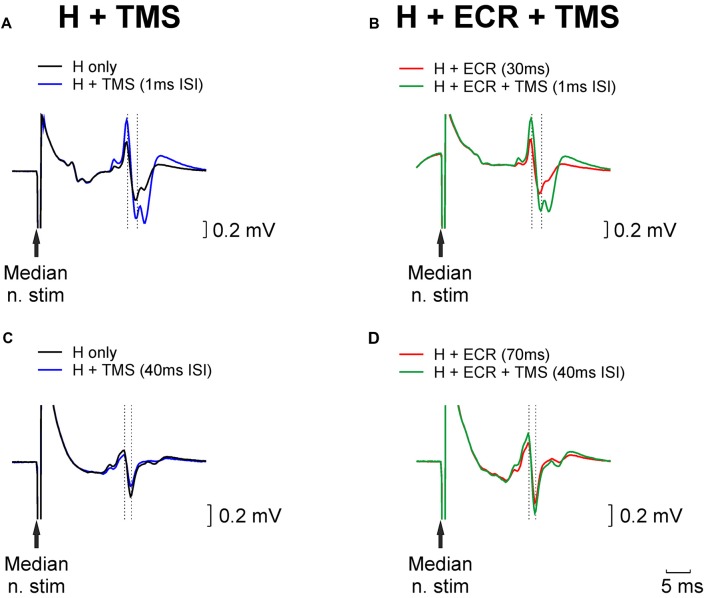
Examples of H reflex conditioning. **(A)** Black trace illustrates an unconditioned H reflex. Blue trace shows the H reflex conditioned by TMS delivered 1 ms before the median nerve stimulus; a clear facilitation is evident. **(B)** Red trace shows the H reflex conditioned by extensor carpi radialis (ECR) stimulation 30 ms before the median nerve stimulus. This facilitated the reflex; the median nerve intensity was reduced until the reflex amplitude approximately matched the unconditioned reflex (black trace, Panel **A**). Green trace illustrates the effect of conditioning this reflex with TMS (same timing relative to median nerve as in Panel **A**). **(C,D)** As for **(A,B)**, but now illustrating ECR stimulation delivered 70 ms before the median nerve stimulus, which produced a reflex suppression, necessitating an increase in stimulus intensity to match the reflex amplitude. TMS in **(C,D)**, was delivered 40 ms before the median nerve stimulus.

Figure 1 shows example traces from a single subject who participated in the TMS experiment. Figure 1A illustrates how the H reflex (black trace) was facilitated by an appropriately-timed TMS pulse (blue trace). Figure 1B (red trace) shows an H reflex which has been facilitated by conditioning by ECR stimulation 30 ms before the median nerve shock which elicited the H reflex. The strength of the median nerve stimulation was reduced, until this facilitated H reflex was approximately the same size as the unconditioned H reflex (compare with the black trace of Figure 1A). When the reflex conditioned by ECR stimulation was further conditioned by TMS (green trace, Figure 1B), with the same interval as used in Figure 1A, it was facilitated. However, this facilitation was smaller than that produced by TMS on the H reflex alone. This indicates that the circuit generating reflex facilitation following ECR stimulation is suppressed by TMS.

Figures 1C,D presents similar traces, for the H reflex suppression produced by conditioning with ECR stimulation 70 ms before the median nerve shock. In this case, the chosen TMS-median nerve interval slightly suppressed the H reflex (compare black and blue traces, Figure 1C). The suppressed H reflex conditioned by the ECR stimulus (red trace, Figure 1D) was matched in amplitude to the unconditioned H reflex (black trace, Figure 1C) by increasing the median nerve stimulus strength. When this reflex was further conditioned by TMS, it was facilitated (compare green and red traces in Figure 1D). This indicates that the circuit generating reflex suppression following ECR stimulation was also suppressed by TMS.

**Figure 2:**
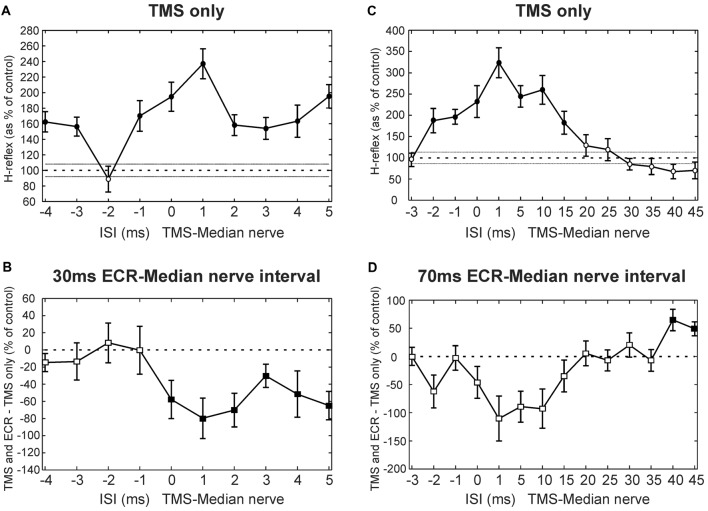
Transcranial magnetic stimulation (TMS) results from single subjects. **(A)** Effects of TMS on the flexor carpi radialis (FCR) H-reflex at different inter-stimulus intervals (ISIs) from a single subject. **(B)** Difference between the effect of TMS on the H reflex, and the effect of TMS on the H reflex conditioned by ECR stimulation 30 ms before the median nerve shock. Same subject as **(A)**. **(C)** Effects of TMS on the FCR H-reflex, for different ISIs and a different subject from **(A)**. **(D)** Difference between effect of TMS on H reflex, and on H reflex conditioned by ECR stimulation 70 ms before the median nerve shock. Same subject as **(D)**. In **(A,C)**, the peak-peak amplitude of the H reflex is plotted as a function of ISI, as a percentage of the size of the unconditioned H reflex. Filled symbols show points significantly different from 100% **(A,C)** or 0% **(B,D)**. Error bars indicate standard error of the mean.

Figure 2 shows results at multiple intervals from a single subject who participated in the TMS experiment. Figure 2A shows how the H reflex was facilitated by TMS in this subject at different ISIs. There was a main effect of ISI (ANOVA, *p* < 0.001). *Post hoc* *t*-tests showed that TMS caused facilitation of the H-reflex at ISIs −4, −3, −1, 0, 1, 2, 3, 4 and 5 ms (all *p* < 0.002; shown with filled circles in Figure 2A). Figure 2B shows, for the same subject, the difference between the effect of TMS on the H reflex conditioned by ECR stimulation with a 30 ms interval, and TMS on the H reflex alone. There was a main effect of both ISI and ECR conditioning stimulation (ANOVA, both *p* < 0.001). *T-tests* showed that the effect of TMS on the H-reflex conditioned by ECR stimulation was significantly smaller than the effect of TMS on the H reflex alone at ISIs 0–5 ms (all *p* < 0.05, Figure 2B).

Figure 2C shows the effect of TMS on the H reflex in a different subject. Again, there was a main effect of ISI (ANOVA, *p* < 0.001), and *post hoc t*-tests showed that TMS facilitated the H-reflex at ISIs −2, −1, 0, 1, 5, 10 and 15 ms (all *p* < 0.005). Figure 2D shows, in this subject, the difference between the effect of TMS on the H reflex and on the H reflex conditioned by ECR stimulation at a 70 ms interval (ECR precedes median nerve). Once again, there was a main effect of ISI and ECR stimulation (ANOVA, all *p* < 0.007). *Post hoc t*-tests indicated that TMS increased the H-reflex conditioned by ECR stimulation more than the H reflex alone at ISIs 40 and 45 ms (all *p* < 0.009, Figure 2D).

**Figure 3:**
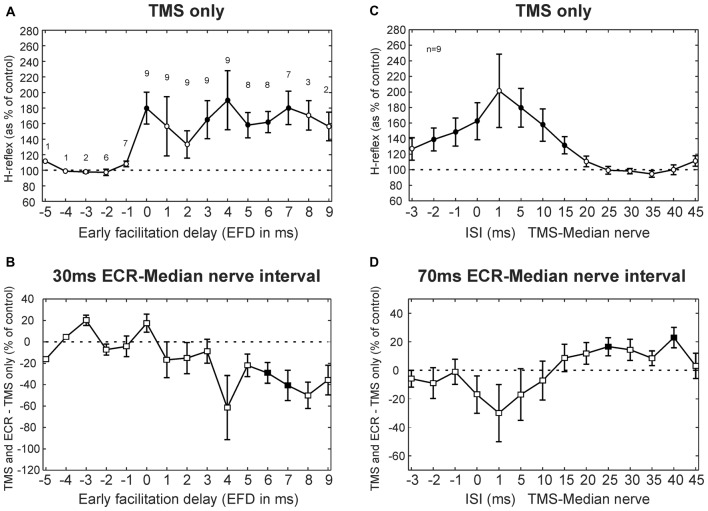
TMS results averaged across subjects. **(A)** Effects of TMS on the FCR H-reflex at different early facilitation delays (EFD). Numbers above each result display the number of subjects contributing to each data point. **(B)** Difference between the effect of TMS on the H reflex, and the effect of TMS on the H reflex conditioned by ECR stimulation 30 ms before the median nerve shock. Numbers of subjects contributing at each interval are as in **(A)**. **(C)** Effects of TMS on the FCR H-reflex at different ISIs. **(D)** Difference between effect of TMS on H reflex, and on H reflex conditioned by ECR stimulation 70 ms before the median nerve shock. Filled symbols show responses significantly different from 100% **(A,C)** or 0% **(B,D)**. Error bars indicate standard error of the mean. **(C,D)** are both averaged over *n* = 9 subjects.

Results averaged across subjects for the TMS experiment are displayed in Figure 3, following a similar layout to the single subject plots of Figure 2. Figure 3A shows how TMS affected the H reflex. The abscissa here is plotted as EFD. As expected, the H reflex for ISIs before 0 ms EFD showed no modulation, but after this interval there was facilitation (main effect of ISI, ANOVA *p* = 0.038; *post hoc t*-tests showed significant facilitation at 0, 3–7 ms, all *p* < 0.047, filled symbols in Figure 3A). The 0 ms EFD occurred at ISIs −4 to 1 ms in the nine subjects tested (−2.1 ± 1.5 ms, mean ± SD). Figure 3B shows the difference between the effect of TMS on the H reflex alone and on the H reflex conditioned by ECR stimulation 30 ms before the median nerve stimulus. ANOVA showed a main effect of factors EFD and ECR conditioning stimulation (all *p* < 0.002). *Post hoc* *t*-tests indicated that TMS had a significantly smaller effect on the H-reflex conditioned by ECR stimulation than on the H reflex alone, for EFDs 6 and 7 ms (all *p* < 0.045, filled symbols, Figure 3B).

Figures 3C,D show TMS results for the 70 ms ECR-Median nerve interval; in this case, the abscissa shows raw TMS-median nerve interval, uncorrected for EFD. TMS produced a broad facilitation of the H reflex (Figure 3C; ANOVA main effect of ISI, *p* < 0.001; *post hoc t*-tests indicated facilitation at ISIs −2, −1, 0, 5, 10 and 15 ms, all *p* < 0.031). Figure 3D shows the difference between the effect of TMS on the H reflex alone, and on the H reflex conditioned by ECR stimulation 70 ms before the median nerve shock. ANOVA showed a main effect of ISI and ECR conditioning stimulation (all *p* < 0.001). *Post hoc t*-tests indicated that TMS had a greater effect on the H-reflex conditioned by ECR stimulation than on the H-reflex alone, for ISIs 25 and 40 ms (all *p* < 0.032, Figure 3D).

In summary, with appropriate timing TMS can significantly decrease the facilitation of the H-reflex by stimulation of the ECR 30 ms before the median nerve shock. TMS is also capable of reducing the suppression of the H-reflex following ECR stimulation 70 ms before the median nerve shock. This suggests that both circuits mediating effects of ECR stimulation on the H reflex are inhibited by corticospinal output activated by TMS.

### Click Experiments

**Figure 4:**
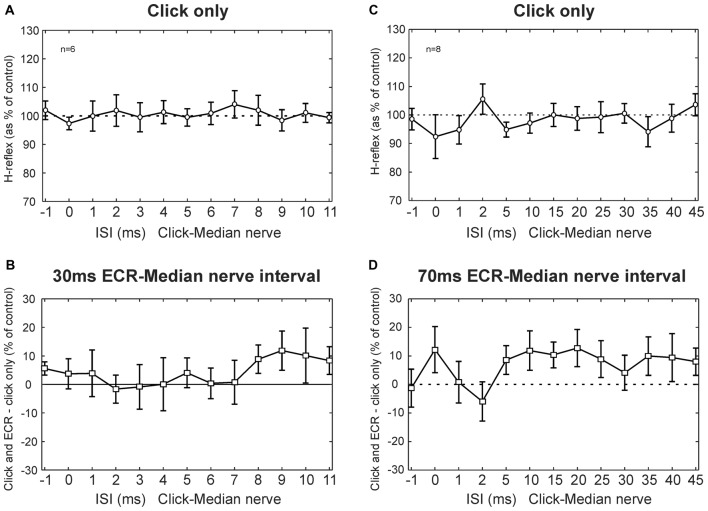
Clicks results averaged across subjects. **(A)** Effects of clicks on the FCR H-reflex at different ISIs. **(B)** Between the effect of click on the H reflex, and the effect of click on the H reflex conditioned by ECR stimulation 30 ms before the median nerve shock which elicited the H reflex. **(A,B)** are averaged over *n* = 6 subjects. **(C)** Effects of clicks on the FCR H-reflex at different ISIs (note different time scale from **A**). **(D)** Difference between effect of click on H reflex, and on H reflex conditioned by ECR stimulation 70 ms before the median nerve shock. **(C,D)** are averaged over *n* = 8 subjects. In no cases in any plot were responses significantly different from control. Error bars indicate standard error of the mean.

Figure 4 shows results from clicks, averaged across subjects. Clicks did not produce a change in the H-reflex amplitude (no main effect of ISI, ANOVA *p* > 0.05; Figure 4A). Likewise, clicks did not produce significantly different effects on the H reflex conditioned by ECR stimulation at 30 ms interval compared with H reflex alone (ANOVA, *p* > 0.05 for both ISI and ECR conditioning, Figure 4B).

Figures 4C,D show results for the 70 ms ECR-Median nerve interval, in a similar format. For the longer intervals used in this experiment, clicks also did not exert an effect on the H reflex (ANOVA, no main effect of ISI, *p* > 0.05, Figure 4C). There was no significant difference between the effect of clicks on the H reflex or on the H reflex conditioned by ECR stimulation (ANOVA, no main effect of ISI or ECR conditioning stimulation, *p* > 0.05, Figure 4D).

Although no significant effects were observed, we were concerned that we might have failed to detect small effects due to statistical thresholding. In particular, it appeared that both Figures 4B,D had groups of points which lay consistently above 100%, even though the error bars were large. To check for this, we repeated the analysis by grouping together sets of three ISIs, with the aim of decreasing the variability and increasing the chances of obtaining significant differences. Yet even with this manipulation, there were no significant differences between the effects of clicks on the H reflex and on the H reflex conditioned by ECR stimulation, for either the 30 ms or 70 ms intervals (*p* > 0.05). Although it is impossible to demonstrate that there is no effect of a particular pathway, any effects must be very weak, failing to be detected even with additional data averaging. We therefore conclude that pathways activated by clicks provide negligible inputs to the circuits mediating facilitation or suppression of the FCR H-reflex following ECR stimulation.

## Discussion

The purpose of this study was to investigate possible descending inputs, from the corticospinal and reticulospinal tracts, to the spinal circuits generating facilitation of the FCR H-reflex at 30 ms and suppression at 70 ms ECR-median nerve interval. Our results suggest that there is negligible input to both circuits from reticulospinal pathways activated by clicks. By contrast, corticospinal circuits activated by TMS appear to inhibit both the facilitation at 30 ms and the suppression at 70 ms ECR-median nerve intervals.

### Corticospinal Tract

**Figure 5:**
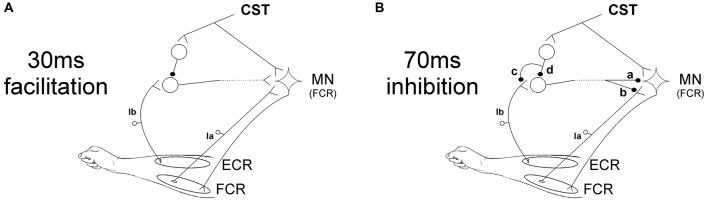
Schematic representation of spinal circuits. **(A)** Corticospinal tract (CST) input to spinal circuit generating facilitation of the FCR H-reflex at 30 ms ECR-Median nerve interval. The CST inhibits Ib interneurons which excite FCR motorneurons (MNs). **(B)** CST input to spinal circuit generating suppression of the FCR H-reflex at 70 ms ECR-Median nerve interval. The components of this circuit are uncertain, and several possibilities are shown. The interneuron excited by Ib afferents may inhibit the FCR motorneurons post-synaptically (a) or pre-synaptically (b). The CST may inhibit this interneuron either via pre-synaptic inhibition to its Ib inputs (c) or via post-synaptic inhibition of the interneuron (d).

The facilitation caused by ECR stimulation 30 ms before the median nerve shock was significantly reduced by TMS at 6 and 7 ms EFDs. Figure 5A shows a schematic representation of the spinal circuit described by Aguiar and Baker (2018) with the addition of CST input to the circuit, which would explain our experimental findings. ECR Ib afferents synapse with Ib interneurons which ultimately excite FCR motorneurons. Although this is shown in Figure 5A as a monosynaptic connection, we cannot tell definitively how many interneurons are involved, or whether this circuit is confined to the spinal segment or includes a non-segmental pathway. However, the earliest effect of TMS was detected at an EFD of 6 ms (Figure 2B). It has been suggested that Ib extensor afferents facilitate flexor motoneurons via a trisynaptic pathway, i.e., with two interposed interneurons (Eccles et al., 1957; Pierrot-Deseilligny and Burke, 2005). The longer effective conditioning interval using TMS is consistent with this, and may indicate that corticospinal inhibitory input is provided to the first, but not the second, of the two interneurons. Another possibility that cannot be ruled out is pre-synaptic inhibition of Ib terminals activated by the CST (as suggested in option c of Figure 5B for the 70 ms inhibition circuit), which would reduce input to the interneurons.

The pathway producing inhibition at 70 ms ECR-Median nerve interval is more uncertain. We have previously speculated (Aguiar and Baker, 2018) that the origin of this suppression might be pre-synaptic at the FCR Ia afferent terminals, comparable to the D2 inhibition seen when conditioning the FCR H-reflex with radial nerve stimulation at intervals of 50–1000 ms (Berardelli et al., 1987). In Figure 5B a schematic representation of the possible spinal circuits generating inhibition is presented, including where CST inputs could contribute. Our results showed that the corticospinal input to the circuit reduces the suppression seen at 70 ms ECR-Median nerve interval when TMS is applied with ISIs of 25 and 45 ms. Figure 5B shows that inhibition of the FCR response could occur either post-synaptically (synapse labeled “a” in the figure) or pre-synaptically (“b”). The interneuron responsible for either pathway must be inhibited by the CST, either pre- (“c”) or post-synaptically (“d”).

The possibility of pre-synaptic inhibition mediating the 70 ms inhibition (“b” in Figure 5B) could be in line with our observation that this 70 ms inhibition is weak or absent in stroke survivors (Aguiar et al., unpublished observations). Loss of inhibition related to changes in KCC2 function are present in spasticity, which is a common consequence of stroke (Toda et al., 2014). We showed weak or absent inhibition at 70 ms ECR-Median nerve interval in 17 stroke patients with spasticity levels 0–3 in the Ashworth scale, although no significant correlation was found between response size and spasticity level (Aguiar et al., unpublished observations) Reduced pre-synaptic inhibition of FCR Ia terminals has been previously demonstrated in hemiplegic patients after stroke (Nakashima et al., 1989). The CST has been shown both to facilitate and suppress pre-synaptic inhibition of Ia terminals (Meunier and Pierrot-Deseilligny, 1998; Meunier, 1999). Our results indicate that the suppression following ECR stimulation is reduced by TMS, indicating an inhibitory input to this circuit activated from the CST.

Several other systems generating inhibition have been described in the literature; any of these could be related to the 70 ms inhibition caused by ECR stimulation. Jenner and Stephens (1982) reported the cutaneomuscular reflex responses following digital nerve stimulation. There is an inhibitory component to this reflex (referred to as the I1 reflex); this appears to be generated by a spinal pathway under descending control, as the I1 is absent in patients with motor cortical damage. The D2 inhibition already described above is another inhibitory system of the motor system, probably of pre-synaptic origin (Berardelli et al., 1987). Other important inhibitory systems include short- (SAI) and long-latency afferent inhibition (LAI), although current knowledge about these circuits is still insufficient, especially in the case of LAI (Turco et al., 2018). The inhibition described here at 70 ms ECR-Median nerve interval might be related to one or more of these previously-described inhibitory systems, including cortical components and afferent inputs. Further work is necessary to uncover the mechanisms involved in this inhibition.

One possible limitation of our study is the fact that some of the individual results showed facilitation of the FCR H-reflex with the earliest ISI tested (−4 ms, as in the example illustrated in Figure 2A). This raises the possibility that the earliest facilitation of the H-reflex actually occurred even earlier, at an interval which we did not test. However, this is unlikely; in the literature, the earliest effect of TMS on the FCR H-reflex is not shorter than −4 ms (Baldissera and Cavallari, 1993; Gracies et al., 1994; Mercuri et al., 1997); shorter intervals would be hard to reconcile with the known conduction delays.

### Reticulospinal Tract

Results from experiments using clicks failed to show any significant effects. First, click sounds alone did not change the FCR H-reflex at any of the ISIs investigated in our experiments. Although there are known reticulospinal inputs to forearm flexor motoneurons in primates, they have an amplitude around five times smaller than inputs from the CST (Riddle et al., 2009). In addition, part of the reticulospinal input comes via a disynaptic pathway, raising the possibility that it may be gated out in the resting conditions tested in our study (Schepens and Drew, 2006). The primate study of Fisher et al. (2012) reported late responses to TMS in reticular formation cells which seemed to be mediated by the click sound generated by the coil discharge. Fisher et al. (2012) suggested that at least part of the click-evoked inputs to reticulospinal cells originated in the vestibular system, which is known to respond to loud clicks (Didier and Cazals, 1989; McCue and Guinan, 1994; Murofushi et al., 1995; Zhu et al., 2011) and to provide inputs to reticulospinal cells (Peterson and Abzug, 1975). In addition, a more conventional pathway involving cochlear nerve inputs to reticulospinal cells is also likely (Nodal and López, 2003). However, out of eight reticulospinal cells in Fisher et al.’s (2012) study, only three responded at the latency consistent with being generated by clicks. It is thus perhaps unsurprising that no statistically significant effects on the H reflex were observed from the partial activation of an already weak input.

Second, we could not detect an effect of the click-evoked activity on either the facilitation or suppression generated by ECR stimulation. Once again, this could be because inputs were too weak to be detected. However, Riddle and Baker (2010) reported that 66% vs. 54% of cervical spinal cord interneurons responded to a train of three corticospinal vs. reticulospinal stimuli, with 0.48 vs. 0.67 extra spikes elicited per stimulus respectively. The strength of inputs from the two descending pathways to interneurons in general is therefore not greatly different. The fact that no inputs could be detected to the circuits tested may suggest that there is a genuine difference here, and that these circuits receive corticospinal, but no reticulospinal inputs. This is possible given the data of Riddle and Baker (2010), who found that of cells which responded to at least one of the descending inputs tested, 15% responded only to stimulation of the medial longitudinal fasciculus (which contains mainly reticulospinal fibers).

Since reticulospinal cells receive input from neck proprioceptors and are therefore influenced by posture (Baker, 2011; Baker et al., 2015), one possible limitation of our study could be that the posture used here might not have been the most appropriate to activate the reticulospinal tract using the click sounds strongly. Testing the protocol with different postures using neck rotations might be appropriate to investigate this possibility further (Ziemann et al., 1999; Tazoe and Perez, 2014).

Another important aspect of our results is that it is uncertain which parts of the reticular formation is activated by the click sounds used in our experiments. Fisher et al. (2012) recorded click responses from cells in the primate nucleus gigantocellularis, which is an important source of the reticulospinal tract (Sakai et al., 2009). In humans, loud clicks comparable to the ones applied here were used in a paired-pulse plasticity protocol, with results consistent with activation of the reticular formation (Foysal et al., 2016), although the exact nucleus involved could not be determined. It remains possible, therefore, that there may be an input from reticulospinal fibers to the circuits investigated here, but that they originate from a region which is not activated by clicks.

## Author Contributions

SNB designed the study. SAA collected the experimental data. SNB and SAA analyzed the results and participated in the production and revision of the manuscript.

## Conflict of Interest Statement

The authors declare that the research was conducted in the absence of any commercial or financial relationships that could be construed as a potential conflict of interest.
